# Synaptic Plasticity and Learning in Animal Models of Tuberous Sclerosis Complex

**DOI:** 10.1155/2012/279834

**Published:** 2012-07-15

**Authors:** Timo Kirschstein

**Affiliations:** Oscar Langendorff Institute of Physiology, University of Rostock, Gertrudenstrasse 9, 18057 Rostock, Germany

## Abstract

Tuberous sclerosis complex (TSC) is caused by a mutation of either the *Tsc1* or *Tsc2* gene. As these genes work in concert to negatively regulate the mammalian target of rapamycin (mTOR) kinase which is involved in protein translation, mutations of these genes lead to a disinhibited mTOR activity. Both the clinical appearance of this condition including tumors, cognitive decline, and epileptic seizures and the molecular understanding of the mTOR signaling pathway, not only involved in cell growth, but also in neuronal functioning, have inspired numerous studies on learning behavior as well as on synaptic plasticity which is the key molecular mechanism of information storage in the brain. A couple of interesting animal models have been established, and the data obtained in these animals will be discussed. A special focus will be laid on differences among these models, which may be in part due to different background strains, but also may indicate pathophysiological variation in different mutations.

## 1. Introduction

Tuberous sclerosis complex (TSC) is an inherited disease caused by a heterozygous germ line mutation of either the *Tsc1* or *Tsc2* gene that is manifested in early childhood. The pathological hallmark of this disorder is the development of hamartomas (benign tumors) arising in a number of organs including the central nervous system [1, 2]. In the brain, TSC lesions typically comprise of cortical tubers, subependymal nodules, and giant cell astrocytomas [3, 4].Hence, common symptoms related to brain lesions are epileptic seizures, mental retardation, multiple neuropsychological impairments, and even autism [5–9]. Consequently, the significant neuropsychiatric morbidity caused by this condition has inspired a number of groups worldwide to study the underlying pathomechanisms aiming to improve our functional understanding of both gene products, named hamartin (*Tsc1*) and tuberin (*Tsc2*). These proteins act in concert as a guanosine triphosphate-activating protein (GAP) towards the small G protein Rheb, which is the key regulator of the mammalian target of rapamycin (mTOR) signaling [10, 11]. Since hamartin and tuberin negatively regulate mTOR activity, which in turn phosphorylates and thereby activates important translation factors such as p70 S6 kinase 1 (S6K1) and eukaryote initiation factor 4E-binding protein (eIF4E-BP), a major role of the TSC-mTOR signaling pathway has been suggested for tumorigenesis, and both genes were initially recognized as tumor suppressors [12]. However, increasing evidence has been provided that this pathway is also considerably involved in neuronal functioning including synaptic plasticity [13–16].

Since its discovery in the early 1970s [17], a large body of evidence has been emerged supporting the key role of synaptic plasticity in memory formation at the molecular level [18, 19]. In general, synaptic plasticity is an activity-dependent change of synaptic strength which appears bidirectional. A rise in synaptic strength or long-term potentiation (LTP) is typically induced by high-frequency stimulation (>50 Hz) paradigms that are capable to activate postsynaptic NMDA receptors [19]. In contrast, long-term depression (LTD) may be a consequence of stimulation paradigms delivered at lower frequencies (1–3 Hz; [20]) or involving the activation of metabotropic glutamate receptors (mGluRs; [21–25]).

## 2. Synaptic Plasticity in TSC Animal Models

Several animal models of TSC are available and have already been employed to study synaptic plasticity in vitro. One unique animal model is the Eker rat, which carries a spontaneous germ line mutation of the *Tsc2* gene and was initially described as a model for renal cancer [37].Only mild brain pathologies were observed in *Tsc*2^+/−^ rats, albeit may be more pronounced in aged animals, and—in addition—epileptic seizures were not detected at all [26, 38–40], (Table 1). However, a “second hit” insult such as irradiation was effective in this model both in lowering seizure threshold and in producing morphological brain lesions [41]. In particular, these included cytomegalic neurons and giant astrocyte-like cells in the cortex, but also in other brain regions such as the hippocampus. With respect to learning disabilities, it is conceivable that cognitive dysfunction may be related to both cortical and hippocampal lesions. Hence, it should be mentioned that the hippocampus of irradiated *Tsc*2^+/−^ rats exhibited multiple nodular hamartoma-like lesions with eosinophilic large cells. Since these cells were immunoreactive for glial fibrillary acidic protein (GFAP), but also for MAP2 and nestin, they seem to share both immature neuronal and glial/astrocytic features [41]. The appearance of such cells within the brain of adult irradiated as well as aged *Tsc*2^+/−^ rats [40, 41] may reflect abnormal processes of cell migration and differentiation during corticogenesis. Synaptic plasticity, unfortunately, has not been tested in these animals. The first attempt to assess synaptic function in a TSC model was made in young naïve *Tsc*2^+/−^ rats [27]. Notably, despite the absence of a clear neuropathology and seizures, synaptic plasticity in the hippocampus was impaired in young naïve *Tsc*2^+/−^ rats [27]. In this early report, a rather physiological theta-burst stimulation paradigm (10 trains of 5 stimuli at 100 Hz, 200 ms apart) was applied to Schaffer collateral-CA1 synapses, which was sufficient to induce robust LTP in control animals. In mutated rats, however, this paradigm was no longer able to produce significant LTP at these synapses. Importantly, the same results were obtained when a GABA_A_ receptor blocker was present in the bathing solution, indicating that the LTP deficit in the Eker rat model was due to *Tsc2* deficiency in CA1 pyramidal neurons rather than in GABAergic interneurons in this region. Moreover, long-term depression induced by two different low-frequency stimulation protocols (900 stimuli at 1 Hz, 2 × 600 stimuli at 1 Hz) was also significantly diminished in *Tsc*2^+/−^ tissue. These findings suggested that mental retardation and cognitive decline in TSC may be due to impaired synaptic plasticity rather than resulting from brain lesions or epileptic seizures.

However, the assumption that cognitive malfunction in TSC may be due to an alteration of activity-dependent synaptic plasticity, in particular at hippocampal synapses, is plausible, but still controversial. The gene products of *Tsc1/2* act in concert in order to downregulate mTOR function [10, 11], therefore TSC is generally associated with a disinhibited activity of this important regulatory enzyme leading to increased protein translation [42, 43]. Just recently, Stoica et al. [44] have demonstrated that mTOR is indeed required for normal synaptic plasticity and long-term memory. They created heterozygous *mTOR* ^+/−^ mice, which themselves did not show altered LTP or a memory deficit phenotype. However, when hippocampal slices taken from these animals were pretreated with low concentrations of rapamycin in order to inhibit the remaining activity of mTOR, LTP was significantly reduced [44]. Moreover, the in vivo administration of low-dose rapamycin to *mTOR* ^+/−^ mice impaired contextual long-term fear memory. Importantly, the low concentration of rapamycin was subthreshold to affect LTP or long-term memory in wild-type mice, indicating that regular mTOR activity is instrumental for hippocampus-dependent forms of long-term memory.

On the neuropathological level, mTOR disinhibition in radial glia led to cortical and hippocampal dyslamination with hippocampal heterotopias and dysplastic neurons [45]. This is an extremely interesting animal model because—like in the human condition—all cells were heterozygous for the *Tsc2* deletion, and radial glia additionally lacked the second copy of the *Tsc2* gene. Unfortunately, this animal model has not yet been tested electrophysiologically. Nonetheless, it is an intriguing question whether *Tsc2* deletion and thereby disinhibited mTOR function affects learning and memory. In particular, it has been demonstrated that mTOR-dependent protein synthesis is required for late phases of LTP [46]. To assess the role of TSC-associated mTOR disinhibition in this form of LTP, heterozygous *Tsc*2^+/−^ mice were used to record long-term potentiation in the CA1 subfield lasting for several hours [29]. Using in vitro brain slices, repetitive trains of tetanic stimulations are typically required to induce such a late phase LTP [47, 48]. However, a single train of tetanic stimulation (100 stimuli at 100 Hz), which failed to induce late phase LTP in wildtype mice as expected, elicited abnormally high levels of potentiation in *Tsc*2^+/−^ mice-up to four hours following tetanization [29]. In contrast to the study of von der Brelie et al. [27], early LTP (i.e., 60 minutes of followup) was not different between both genotypes. Unfortunately, different stimulation paradigms (theta-burst versus tetanization) and different animal models (Eker rat versus *Tsc2* knockout mouse) impede a conclusive comparison of both studies. Nonetheless they suggest that constitutively disinhibited mTOR activity and thereby increased protein synthesis in hippocampal neurons significantly enhanced the propensity of late phase LTP, while early phase LTP, which depends on posttranslational modifications such as AMPA receptor phosphorylation via Ca^2+^/calmodulin-dependent kinase II and protein kinase A [49, 50] rather than protein synthesis, is impaired in TSC2 animal models.

With respect to *Tsc1* gene mutations, one interesting animal model is the *Tsc*1^GFAP^ conditional knockout mouse, in which the *Tsc1* gene was specifically inactivated in glia [51, 52]. Consequently, the gene product hamartin was absent in brain glia and the mice showed an increased astrocyte proliferation with abnormal neuronal organization, as well as frequent and severe seizures [51]. The detailed pathological examination of adult *Tsc*1^GFAP^ mice revealed that cortical and hippocampal astrocytes expressed vimentin and brain lipid binding protein (BLBP) [52], which are marker proteins for radial glia and immature astrocytes [53, 54]. This alteration was observed throughout the hippocampus including all Cornu Ammonis subfields as well as the dentate gyrus. Hence, the authors concluded that *Tsc1* inactivation in glial cells may impair hippocampal maturation [52]. Interestingly, *Tsc1* ablation in neurons achieved by engineering mice that have a specific *Tsc1* deletion in synapsin-expressing neurons (*Tsc*1^Synapsin^ mice) [55] primarily showed ectopic enlarged and/or dysplastic neurons in the cortex and the hippocampus. Zeng et al. [34] assessed synaptic plasticity in *Tsc*1^GFAP^ animals and—again, LTP at the Schaffer collateral—CA1 synapses was tested. Despite a quite strong induction protocol consisting of four trains of tetanic stimulations (100 stimuli at 100 Hz), the potentiation after 30 minutes was negligible in the mutant mice. However, the authors also found significantly elevated glutamate levels in *Tsc*1^GFAP^ mutants, and importantly, LTP could be rescued by a low concentration of an NMDA receptor antagonist. The authors concluded that excessive glutamate release caused both excitotoxicity in the hippocampus and impaired synaptic plasticity. Since the functional role of mTOR activity might be quite diverse concerning glia and neurons, it appears difficult to reconcile the findings obtained in the *Tsc*1^GFAP^ conditional knockout mouse with those from animals carrying neuronal *Tsc2* mutations.

In summary, a uniform picture on electrically induced synaptic plasticity in various animal models of TSC might not be drawn from the available studies. On the one hand, this is certainly due to different stimulation paradigms that may evoke distinct LTP mechanisms. Alternatively, discrepancies among studies might also be attributed to previously unknown downstream effects of *Tsc1/2* gene inactivation. For instance, a recent report using a dominant negative *Tsc2* mutant (ΔRG mouse model) observed an elevated activity of extracellular signal-regulated kinase (ERK) [31]. The ERK pathway, however, has not been addressed in the investigations discussed above [27, 29, 34].

A mechanistically distinct form of long-term depression has been discovered in the CA1 stratum radiatum that depends on activation of group I metabotropic glutamate receptors (mGluR-LTD; [21–25]). A large body of evidence suggests that mGluR-LTD is mediated by protein translation-dependent AMPA receptor endocytosis [23–25, 56, 57], involving the mTOR pathway [58, 59] as well as ERK [60]. This notion has inspired three recent investigations on mGluR-LTD: (i) in *Tsc1*-deleted CA1 neurons [35], (ii) in *Tsc*2^+/−^ mice [30], and (iii) in the ΔRG mouse model of TSC2 [31]. In the first paper, the stereotactic injection of a viral vector encoding a Cre recombinase-EGFP construct under the control of the synapsin promoter into the CA1 area of *Tsc*1^flox/flox^ mice [61] provided an extremely elegant method to delete the *Tsc1* gene specifically in CA1 pyramidal neurons [35]. Pharmacological activation of group I mGluRs using the specific agonist (RS)-3,5-dihydroxyphenylglycine (DHPG, 100 *μ*M, 5 min) caused an immediate as well as a long-lasting depression (i.e., mGluR-LTD) of excitatory postsynaptic currents (EPSCs) in CA1 neurons. Both types of depression were also achieved by an electrical stimulation paradigm consisting of 900 pairs of stimuli at 1 Hz (paired-pulse low-frequency stimulation, PP-LFS) in the presence of an NMDA receptor antagonist which has also been referred to as an mGluR-dependent LTD induction protocol [24]. In contrast, synaptic transmission onto neurons that were infected with the viral construct and thus lacked the *Tsc1* gene was immediately depressed after DHPG or PP-LFS, but the induction of LTD was prevented. These findings unequivocally demonstrated that intact *Tsc1* functioning is required for mGluR-LTD in the CA1 area. However, these findings do not explain how disinhibition of mTOR activity and thus enhanced protein synthesis might compromise mGluR-LTD which was initially assumed to be more pronounced in tissue after loss of *Tsc1/2* [62].

A severe reduction of mGluR-LTD was also found in *Tsc*2^+/−^ mice [30]. In this paper, the authors tested both standard induction protocols, that is, brief application of DHPG and PP-LFS, and thus confirmed that heterozygous deletion of the *Tsc2* gene impaired mGluR-LTD. Moreover, protein synthesis inhibition with cycloheximide mimicked this reduction, and had no effect in *Tsc*2^+/−^ mice. On the other hand, the mGluR-LTD was restored by pretreatment with rapamycin, indicating that the impairment was due to excess mTOR activity [30]. The third recent paper again confirmed the loss of mGluR-LTD in dominant negative *Tsc2* mutant mice (ΔRG mice) and found an elevated activity of extracellular signal-regulated kinase (ERK) in these animals [31]. The ΔRG transgenic mouse expresses a dominant negative *Tsc2* gene product which binds to and recruits hamartin, but disables the TSC1/2 protein complex in terms of their Rheb-directed GAP activity [63, 64]. Surprisingly, phosphorylation of both S6K1 and eIF4E-BP remained unaltered indicating that the mTOR signaling pathway was intact. However, the ribosomal S6 protein was detected to be phosphorylated at serine residues specific for ERK1/2 activation. Thus, *Tsc2* inactivation by the ΔRG mutation caused an impairment of mGluR-LTD, which was due to overactivation of ERK1/2, but not of mTOR [31]. The authors also tested transgenic mice in which the *Tsc1* or *Tsc2* genes were specifically deleted in *α*-Ca^2+^/calmodulin-kinase II-expressing neurons. In line with the other two models described above (*Tsc1*-deleted CA1 neurons using viral transfection, and the ΔRG mouse model), these strains again showed the same phenotype that is, impaired DHPG-induced LTD. Hence, from the available literature on mGluR-dependent LTD in the CA1 area, it can be concluded that genetic ablation of either the *Tsc1* or *Tsc2* is consistently associated with the loss of this form of synaptic plasticity.

## 3. Learning and Memory in TSC Animal Models

The manifestation of cognitive decline in TSC patients is ranging from moderate to severe, and even autistic behaviors have been observed [5–9]. To address this issue experimentally, a number of different behavioral test batteries assessing distinct brain functions in rodents have been used to characterize the phenotypes of currently available TSC animal models. The first attempt to study learning behavior and memory in TSC was again made with Eker rats [26]. Although the authors tried hard to obtain a memory-impaired phenotype in young Eker rats that were almost devoid of cerebral hamartomas [26, 38–40], the only significant difference was discovered in a delayed matching-to-place task assessing episodic-like memory [65]. First, the authors tested this specific type of memory task in a water maze, where the hidden platform was kept constant during four trials within the same experimental day, but was randomly repositioned at consecutive days. In the control condition, a trial-to-trial interval of 15 seconds was chosen resulting in no significant difference in the learning behavior between *Tsc*2^+/−^ rats and control animals. In contrast, when the interval of the first to the second trial was prolonged to two hours (i.e., the delayed matching-to-place task), *Tsc*2^+/−^ rats surprisingly showed significantly better performance than control littermates which was confirmed in a radial maze with similar paradigm using a randomized baiting scheme. All other behavioral tests assessing exploration and anxiety (light/dark box) as well as learning and memory (Pavlovian conditioned taste aversion test, Morris water maze hidden platform task including reversal learning, and probe trials) did not detect any behavioral constraints in *Tsc*2^+/−^ rats [26].

Recently, the same group has published a reexamination of the Eker rat model behavioral phenotype—now comparing the naïve animal with an epileptic condition following systemic administration of kainic acid [28]. Again, they did not observe significantly different performance in learning abilities (fear conditioning, fear extinction, and Morris water maze). Thus, they were able to reproduce their previous report, but a somewhat unexpected finding was that kainic acid-injected rats also turned out to behave like controls in these learning tasks. In contrast, rearing in the open field, novel object recognition, and social exploration was in fact significantly reduced in naïve *Tsc*2^+/−^ rats, and even more so in epileptic ones. The authors concluded that both *Tsc2* haploinsufficiency and epileptic seizures might compromise social interaction and moreover, to some extent in an additive manner. Albeit the quantitative differences obtained in these tests occasionally appeared to be less impressive, this is a potentially important issue in the clinical management of the disease.

Mice carrying a heterozygous inactivation of the *Tsc2* gene (*Tsc*2^+/−^ mice) have already been mentioned in Section 2. In the same paper introduced above [29], the authors also looked at hippocampus-dependent learning behavior. First, the animals were allowed to learn the position of a hidden platform in the Morris water maze, then the platform was removed (probe trial), and two measures were used to assess whether the animals have learned the target position. Both the time spent in the quadrant of the prior platform position and the number of target crossings was analyzed and the performance of *Tsc*2^+/−^ mice was found to be significantly poorer than that observed in controls. The second behavioral test (eight-arm radial maze) revealed that significantly more across-phase errors have been committed by the mutant mice. In the last test, the mice were trained in context fear conditioning using an aversive stimulus. 24 hours later, the animals were transferred either back into the training context or into a novel context, and the freezing responses were measured. With this paradigm, *Tsc*2^+/−^ mice showed poor context specificity, that is, freezing in the novel context was not different from freezing in the training context [29].

The dominant negative *Tsc2* transgenic mouse mutant has been also been described in detail in Section 2 [31]. In two studies [32, 33], these mice have been investigated in various behavioral tasks assessing anxiety-related behavior as well as learning tasks. Anxiety-related behavior was tested in the elevated plus maze [32].This is a four-arm maze with two open arms and two arms having walls on the sides. It was mounted 1 m above the floor, and the time spent in the open arms was measured. Generally, increased levels of anxiety are associated with less time spent in these open arms. Indeed, dominant negative *Tsc2* mutants showed increased anxiety in this test. In the open field, *Tsc2* mutant mice showed a trend towards less exploration of the inner sector which may also be attributed to increased anxiety. Hippocampus-dependent learning as assessed by the hidden platform task in the Morris water maze and context discrimination after fear conditioning was only mildly affected. More recently, however, another group [33] failed to reproduce these results, and in fact observed normal Morris water maze behavior as well as normal contextual fear. However, social interaction was impaired in these mice. This is a clinically important issue which may relate to autistic behavior observed in TSC patients [5]. In conclusion, dominant negative *Tsc2* mutant mice showed markedly less impaired learning deficits compared with *Tsc*2^+/−^ mice [29].

Cognitive performance may also be affected by *Tsc1* mutations, and two different models have been evaluated so far. In *Tsc*1^+/−^mice [36], no preference for the target quadrant in the Morris water maze probe trial was found, and context-dependent freezing after fear conditioning was significantly less compared to wildtype littermates. Since both tests depend on intact hippocampal functioning, this study indicates a clear deficit in hippocampus-dependent learning. In addition, it is important to note that these findings were obtained despite the absence of seizures and cerebral lesions resembling the observations made with *Tsc*2^+/−^ (Eker) rats [26, 28]. The findings in *Tsc*1^+/−^ mice were largely confirmed in *Tsc*1^*GFAP*^ conditional knockout mice [34]. Using this model, LTP was found to be impaired-as discussed above [34], and performance in the Morris water maze as well as in context fear conditioning was also affected. Thus, unlike *Tsc2* mouse models [29, 32, 33], *Tsc1* mutations were consistently associated with impaired performance in hippocampus-dependent learning paradigms [34, 36]. Social behavior has been evaluated in *Tsc*1^+/−^ mice [36], and both social interaction as well as nest building behavior was markedly reduced in these animals indicating severe social deficits. While learning disabilities have been found to vary in *Tsc1* and *Tsc2* mutations, social behavior was consistently impaired in two studies which employed two different TSC models [33, 36].

## 4. Conclusions and Future Perspectives

Tuberous sclerosis complex (TSC) is a tumorous disease associated with epileptic seizures and mental retardation, sometimes even leading to autism [1–9]. The cognitive decline in these patients may be related to cortical tumors and/or to the epileptic condition. Albeit the latter has not yet been tested experimentally, there is a large body of both clinical and experimental evidence that hippocampal seizures are a leading cause of cognitive dysfunction [66–69]. Alternatively, there are no ultrastructural studies on synaptic morphology in TSC animal models with respect to the hippocampus. Thus, one can speculate whether altered synaptogenesis is causing deficits in hippocampus-dependent LTP and/or learning behavior. At least in *Tsc1* ^GFAP^ mice, it was observed that glial glutamate transport was impaired due to reduced expression of GLT-1 and GLAST protein [70]. However, the idea that cognitive deficits may arise independently from hamartomas and epileptic discharges is an attractive and yet testable hypothesis. Support for this hypothesis is at least derived from available data on animal models that are devoid of epileptic seizures and cerebral lesions presenting with deficits in synaptic plasticity and/or learning capabilities [26–28, 36]. A second line of evidence for this idea is now emerging: a series of recent papers tried to unravel the role of mTOR in long-term memory. These studies did not primarily focus on TSC pathophysiology, but showed that (i) inhibition of mTOR activity blocked both long-term synaptic plasticity and hippocampus-dependent memory [44], (ii) significantly higher mTOR activation was detected in memory-unimpaired aged rats as opposed to memory-impaired ones [71], and (iii) mTOR was required for long-term memory in mutant mice expressing autophosphorylation-deficient *α*-Ca^2+^/calmodulin-kinase II [72]. Importantly, the latter study also employed electron microscopy and confirmed that behavioral long-term memory formation was accompanied with an increased synaptogenesis [72]. Thus, disinhibited mTOR activation appears to be sufficient for synaptic plasticity, synapse formation, and memory consolidation. Of course, disrupted synaptogenesis within hamartomatous lesions as well as epileptic hyperexcitability cannot be excluded as reasons to explain cognitive decline. Rather, these conditions will have an additional impact on higher cerebral functions, and further data are required to assess their relative contributions.

It is widely accepted that understanding the pathophysiology of TSC on a molecular level will be instrumental in improving the clinical management of this disease. However, although various animal models are available and have already been employed in a number of studies, occasionally considerable differences exist concerning at least synaptic physiology and in vivo behavior. Therefore, unraveling the underlying pathomechanisms of *Tsc1* or *Tsc2* mutations (e.g., with respect to cellular or regional differences) will be required to allow a more predictive correlation between cognitive dysfunction in distinct behavioral paradigms and the causative genotype.

## Figures and Tables

**Table 1:** Overview of available data on synaptic plasticity and memory.

Animal model	Synaptic plasticity	Learning and memory	References
*Ts* *c*2^+/−^ rat (Eker rat)	Reduced theta-burst stimulation-induced early phase LTP at Schaffer collateral-CA1 synapses. Reduced low-frequency stimulation-induced LTD at Schaffer collateral-CA1 synapses.	Enhanced episodic-like memory (water maze, radial maze). Mildly reduced exploration behavior (open field, novel object recognition, social exploration). No difference in anxiety-related behavior (light/dark box). No difference in hippocampus-dependent learning behavior (fear conditioning, water maze).	[26–28]
*Ts* *c*2^+/−^ mouse	Enhanced tetanus-induced late phase LTP at Schaffer collateral-CA1 synapses. No difference in tetanus-induced early phase LTP at Schaffer collateral-CA1 synapses. Reduced mGluR-dependent LTD at Schaffer collateral-CA1 synapses.	Reduced hippocampus-dependent learning behavior (water maze, radial maze, context fear conditioning).	[29, 30]
Dominant negative *Tsc2* mutant mouse (ΔRG mouse)	Reduced mGluR-dependent LTD at Schaffer collateral-CA1 synapses.	Increased anxiety-related behavior (elevated plus maze). Mildly impaired hippocampus-dependent learning behavior (water maze, context fear conditioning). Impaired social interaction.	[31–33]
*Tsc1 * ^GFAP^ CKO mouse	Reduced tetanus-induced early phase LTP at Schaffer collateral-CA1 synapses.	Reduced hippocampus-dependent learning behavior (water maze, context fear conditioning).	[34]
Virus-injected mouse (*Tsc1*-deletion in CA1 neurons)	Reduced mGluR-dependent LTD at Schaffer collateral-CA1 synapses.		[35]
*Ts* *c*1^+/−^ mouse		Reduced hippocampus-dependent learning behavior (water maze, context fear conditioning).	[36]

## References

[1] Crino PB, Nathanson KL, Henske EP (2006). Medical progress: the tuberous sclerosis complex. *The New England Journal of Medicine*.

[2] Curatolo P, Bombardieri R, Jozwiak S (2008). Tuberous sclerosis. *The Lancet*.

[3] Huttenlocher PR, Wollmann RL (1991). Cellular neuropathology of tuberous sclerosis. *Annals of the New York Academy of Sciences*.

[4] Mizuguchi M, Takashima S (2001). Neuropathology of tuberous sclerosis. *Brain and Development*.

[5] Bolton PF, Park RJ, Higgins JNP, Griffiths PD, Pickles A (2002). Neuro-epileptic determinants of autism spectrum disorders in tuberous sclerosis complex. *Brain*.

[6] Joinson C, O’Callaghan FJ, Osborne JP, Martyn C, Harris T, Bolton PF (2003). Learning disability and epilepsy in an epidemiological sample of individuals with tuberous sclerosis complex. *Psychological Medicine*.

[7] Prather P, de Vries PJ (2004). Behavioral and cognitive aspects of tuberous sclerosis complex. *Journal of Child Neurology*.

[8] de Vries P, Humphrey A, McCartney D (2005). Consensus clinical guidelines for the assessment of cognitive and behavioural problems in Tuberous Sclerosis. *European Child and Adolescent Psychiatry*.

[9] Winterkorn EB, Pulsifer MB, Thiele EA (2007). Cognitive prognosis of patients with tuberous sclerosis complex. *Neurology*.

[10] Manning BD, Cantley LC (2003). Rheb fills a GAP between TSC and TOR. *Trends in Biochemical Sciences*.

[11] Tee AR, Manning BD, Roux PP, Cantley LC, Blenis J (2003). Tuberous sclerosis complex gene products, tuberin and hamartin, control mTOR signaling by acting as a GTPase-activating protein complex toward Rheb. *Current Biology*.

[12] Jozwiak J (2006). Hamartin and tuberin: working together for tumour suppression. *International Journal of Cancer*.

[13] Tavazoie SF, Alvarez VA, Ridenour DA, Kwiatkowski DJ, Sabatini BL (2005). Regulation of neuronal morphology and function by the tumor suppressors *Tsc1* and *Tsc2*. *Nature Neuroscience*.

[14] Jaworski J, Sheng M (2006). The growing role of mTOR in neuronal development and plasticity. *Molecular Neurobiology*.

[15] de Vries PJ, Howe CJ (2007). The tuberous sclerosis complex proteins - a GRIPP on cognition and neurodevelopment. *Trends in Molecular Medicine*.

[16] Richter JD, Klann E (2009). Making synaptic plasticity and memory last: mechanisms of translational regulation. *Genes and Development*.

[17] Bliss TVP, Lomo T (1973). Long lasting potentiation of synaptic transmission in the dentate area of the anaesthetized rabbit following stimulation of the perforant path. *Journal of Physiology*.

[18] Bliss TVP, Collingridge GL (1993). A synaptic model of memory: long-term potentiation in the hippocampus. *Nature*.

[19] Kandel ER (2001). The molecular biology of memory storage: a dialogue between genes and synapses. *Science*.

[20] Collingridge GL, Peineau S, Howland JG, Wang YT (2010). Long-term depression in the CNS. *Nature Reviews Neuroscience*.

[21] Palmer MJ, Irving AJ, Seabrook GR, Jane DE, Collingridge GL (1997). The group I mGlu receptor agonist DHPG induces a novel form of LTD in the CA1 region of the hippocampus. *Neuropharmacology*.

[22] Oliet SHR, Malenka RC, Nicoll RA (1997). Two distinct forms of long-term depression coexist in CA1 hippocampal pyramidal cells. *Neuron*.

[23] Huber KM, Kayser MS, Bear MF (2000). Role for rapid dendritic protein synthesis in hippocampal mGluR- dependent long-term depression. *Science*.

[24] Huber KM, Roder JC, Bear MF (2001). Chemical induction of mGluR5- and protein synthesis-dependent long-term depression in hippocampal area CA1. *Journal of Neurophysiology*.

[25] Snyder EM, Philpot BD, Huber KM, Dong X, Fallon JR, Bear MF (2001). Internalization of ionotropic glutamate receptors in response to mGluR activation. *Nature Neuroscience*.

[26] Waltereit R, Welzl H, Dichgans J, Lipp HP, Schmidt WJ, Weller M (2006). Enhanced episodic-like memory and kindling epilepsy in a rat model of tuberous sclerosis. *Journal of Neurochemistry*.

[27] von der Brelie C, Waltereit R, Zhang L, Beck H, Kirschstein T (2006). Impaired synaptic plasticity in a rat model of tuberous sclerosis. *European Journal of Neuroscience*.

[28] Waltereit R, Japs B, Schneider M, de Vries PJ, Bartsch D (2011). Epilepsy and *Tsc2* haploinsufficiency lead to autistic-like social deficit behaviors in rats. *Behavior Genetics*.

[29] Ehninger D, Han S, Shilyansky C (2008). Reversal of learning deficits in a *Tsc2*
^+/-^ mouse model of tuberous sclerosis. *Nature Medicine*.

[30] Auerbach BD, Osterweil EK, Bear MF (2011). Mutations causing syndromic autism define an axis of synaptic pathophysiology. *Nature*.

[31] Chévere-Torres I, Kaphzan H, Bhattacharya A (2012). Metabotropic glutamate receptor-dependent long-term depression is impaired due to elevated ERK signaling in the ΔRG mouse model of tuberous sclerosis complex. *Neurobiology of Disease*.

[32] Ehninger D, Silva AJ (2011). Increased levels of anxiety-related behaviors in a *Tsc2* dominant negative transgenic mouse model of tuberous sclerosis. *Behavior Genetics*.

[33] Chévere-Torres I, Maki JM, Santini E, Klann E (2012). Impaired social interactions and motor learning skills in tuberous sclerosis complex model mice expressing a dominant/negative form of tuberin. *Neurobiology of Disease*.

[34] Zeng LH, Ouyang Y, Gazit V (2007). Abnormal glutamate homeostasis and impaired synaptic plasticity and learning in a mouse model of tuberous sclerosis complex. *Neurobiology of Disease*.

[35] Bateup HS, Takasaki KT, Saulnier JL, Denefrio CL, Sabatini BL (2011). Loss of *Tsc1* in vivo impairs hippocampal mGluR-LTD and increases excitatory synaptic function. *Journal of Neuroscience*.

[36] Goorden SMI, van Woerden GM, van der Weerd L, Cheadle JP, Elgersma Y (2007). Cognitive deficits in *Tsc1*
^+/-^ mice in the absence of cerebral lesions and seizures. *Annals of Neurology*.

[37] Eker R, Mossige J, Vincents Johannessen J, Aars H (1981). Hereditary renal adenomas and adenocarcinomas in rats. *Diagnostic Histopathology*.

[38] Yeung RS, Katsetos CD, Klein-Szanto A (1997). Subependymal astrocytic hamartomas in the Eker rat model of tuberous sclerosis. *American Journal of Pathology*.

[39] Mizuguchi M, Takashima S, Yamanouchi H, Nakazato Y, Mitani H, Hino O (2000). Novel cerebral lesions in the Eker rat model of tuberous sclerosis: cortical tuber and anaplastic ganglioglioma. *Journal of Neuropathology and Experimental Neurology*.

[40] Takahashi DK, Dinday MT, Barbaro NM, Baraban SC (2004). Abnormal cortical cells and astrocytomas in the Eker rat model of tuberous sclerosis complex. *Epilepsia*.

[41] Wenzel HJ, Patel LS, Robbins CA, Emmi A, Yeung RS, Schwartzkroin PA (2004). Morphology of cerebral lesions in the Eker rat model of tuberous sclerosis. *Acta Neuropathologica*.

[42] Chan JA, Zhang H, Roberts PS (2004). Pathogenesis of tuberous sclerosis subependymal giant cell astrocytomas: biallelic inactivation of *Tsc1* or *Tsc2* leads to mTOR activation. *Journal of Neuropathology and Experimental Neurology*.

[43] Crino PB (2004). Molecular pathogenesis of tuber formation in tuberous sclerosis complex. *Journal of Child Neurology*.

[44] Stoica L, Zhu PJ, Huang W, Zhou H, Kozma SC, Costa-Mattioli M (2011). Selective pharmacogenetic inhibition of mammalian target of Rapamycin complex I (mTORC1) blocks long-term synaptic plasticity and memory storage. *Proceedings of the National Academy of Sciences of the United States of America*.

[45] Way SW, Mckenna J, Mietzsch U, Reith RM, Wu HCJ, Gambello MJ (2009). Loss of *Tsc2* in radial glia models the brain pathology of tuberous sclerosis complex in the mouse. *Human Molecular Genetics*.

[46] Tang SJ, Reis G, Kang H, Gingras AC, Sonenberg N, Schuman EM (2002). A rapamycin-sensitive signaling pathway contributes to long-term synaptic plasticity in the hippocampus. *Proceedings of the National Academy of Sciences of the United States of America*.

[47] Frey U, Krug M, Reymann KG, Matthies H (1988). Anisomycin, an inhibitor of protein synthesis, blocks late phases of LTP phenomena in the hippocampal CA1 region in vitro. *Brain Research*.

[48] Huang YY, Kandel ER (1994). Recruitment of long-lasting and protein kinase A-dependent long-term potentiation in the CA1 region of hippocampus requires repeated tetanization. *Learning Memory*.

[49] Lee HK, Barbarosie M, Kameyama K, Bear MF, Huganir RL (2000). Regulation of distinct AMPA receptor phosphorylation sites during bidirectional synaptic plasticity. *Nature*.

[50] Lisman J, Schulman H, Cline H (2002). The molecular basis of CaMKII function in synaptic and behavioural memory. *Nature Reviews Neuroscience*.

[51] Uhlmann EJ, Wong M, Baldwin RL (2002). Astrocyte-specific *Tsc1* conditional knockout mice exhibit abnormal neuronal organization and seizures. *Annals of Neurology*.

[52] Ess KC, Uhlmann EJ, Li W (2004). Expression profiling in tuberous sclerosis complex (TSC) knockout mouse astrocytes to characterize human TSC brain pathology. *Glia*.

[53] Kurtz A, Zimmer A, Schnutgen F, Bruning G, Spener F, Muller T (1994). The expression pattern of a novel gene encoding brain-fatty acid binding protein correlates with neuronal and glial cell development. *Development*.

[54] Hartfuss E, Galli R, Heins N, Götz M (2001). Characterization of CNS precursor subtypes and radial glia. *Developmental Biology*.

[55] Meikle L, Talos DM, Onda H (2007). A mouse model of tuberous sclerosis: neuronal loss of *Tsc1* causes dysplastic and ectopic neurons, reduced myelination, seizure activity, and limited survival. *Journal of Neuroscience*.

[56] Ronesi JA, Huber KM (2008). Homer interactions are necessary for metabotropic glutamate receptor-induced long-term depression and translational activation. *Journal of Neuroscience*.

[57] Waung MW, Pfeiffer BE, Nosyreva ED, Ronesi JA, Huber KM (2008). Rapid Translation of Arc/Arg3.1 Selectively Mediates mGluR-Dependent LTD through Persistent Increases in AMPAR Endocytosis Rate. *Neuron*.

[58] Hou L, Klann E (2004). Activation of the phosphoinositide 3-kinase-Akt-mammalian target of rapamycin signaling pathway is required for metabotropic glutamate receptor-dependent long-term depression. *Journal of Neuroscience*.

[59] Banko JL, Hou L, Klann E (2004). NMDA receptor activation results in PKA- and ERK-dependent Mnk1 activation and increased eIF4E phosphorylation in hippocampal area CA1. *Journal of Neurochemistry*.

[60] Gallagher SM, Daly CA, Bear MF, Huber KM (2004). Extracellular signal-regulated protein kinase activation is required for metabotropic glutamate receptor-dependent long-term depression in hippocampal area CA1. *Journal of Neuroscience*.

[61] Kwiatkowski DJ, Zhang H, Bandura JL (2002). A mouse model of *Tsc1* reveals sex-dependent lethality from liver hemangiomas, and up-regulation of p70S6 kinase activity in *Tsc1* null cells. *Human Molecular Genetics*.

[62] Kelleher RJ, Bear MF (2008). The autistic neuron: troubled translation?. *Cell*.

[63] Govindarajan B, Brat DJ, Csete M (2005). Transgenic expression of dominant negative tuberin through a strong constitutive promoter results in a tissue-specific tuberous sclerosis phenotype in the skin and brain. *Journal of Biological Chemistry*.

[64] Pasumarthi KBS, Nakajima H, Nakajima HO, Jing S, Field LJ (2000). Enhanced cardiomyocyte DNA synthesis during myocardial hypertrophy in mice expressing a modified *Tsc2* transgene. *Circulation Research*.

[65] Steele RJ, Morris RGM (1999). Delay-dependent impairment of a matching-to-place task with chronic and intrahippocampal infusion of the NMDA-antagonist D-AP5. *Hippocampus*.

[66] Reid IC, Stewart CA (1997). Seizures, memory and synaptic plasticity. *Seizure*.

[67] Beck H, Goussakov IV, Lie A, Helmstaedter C, Elger CE (2000). Synaptic plasticity in the human dentate gyrus. *Journal of Neuroscience*.

[68] Kirschstein T, Bauer M, Müller L (2007). Loss of metabotropic glutamate receptor-dependent long-term depression via downregulation of mGluR5 after status epilepticus. *Journal of Neuroscience*.

[69] Sgobio C, Ghiglieri V, Costa C (2010). Hippocampal synaptic plasticity, memory, and epilepsy: effects of long-term valproic acid treatment. *Biological Psychiatry*.

[70] Wong M, Ess KC, Uhlmann EJ (2003). Impaired glial glutamate transport in a mouse tuberous sclerosis epilepsy model. *Annals of Neurology*.

[71] Ménard C, Quirion R (2012). Successful cognitive aging in rats: a role for mGluR5 glutamate receptors, homer 1 proteins and downstream signaling pathways. *PLoS ONE*.

[72] Radwanska K, Medvedev NI, Pereira GS (2011). Mechanism for long-term memory formation when synaptic strengthening is impaired. *Proceedings of the National Academy of Sciences of the United States of America*.

